# Loss of Tooth Structure After Successive Removal of Temporary Restorative Materials by Dental Students Between Endodontic Treatment Sessions: An In Vitro Study

**DOI:** 10.1111/eje.70083

**Published:** 2025-12-19

**Authors:** Márcia Cunha, Andressa Galzer dos Santos, Roberto Zimmer, Fernanda Zanella Arruda, Guilherme Anziliero Arossi, Fernando Freitas Portella

**Affiliations:** ^1^ Universidade Feevale Novo Hamburgo RS Brazil; ^2^ University of Maryland School of Dentistry Baltimore Maryland USA

**Keywords:** endodontic treatment, glass ionomer cement, linear regression, temporary restoration, zinc oxide

## Abstract

**Objective:**

The aim of this study was to evaluate the loss of tooth structure after the removal of temporary restorative materials between endodontic treatment sessions performed by undergraduate students.

**Methods:**

Twenty human molar teeth underwent endodontic coronal access and were randomly divided into two groups. One group received a temporary restoration with zinc oxide and calcium sulfate‐based temporary cement (ZNO) alone, while the other group was restored with a combination of zinc oxide and calcium sulfate‐based cement at the base of the restoration, and conventional glass ionomer cement as the outer layer (GIC). Every week, four trained undergraduate dental students performed the coronal reopening of the teeth. The teeth were individually weighed after the coronal access and after each removal of the temporary restoration. This process was carried out for 4 weeks. After the fourth week, two specialists in restorative dentistry fully removed any remaining temporary materials and finished the cavity.

**Results:**

The average tooth mass variation was calculated at each removal stage. Linear regression analysis was performed to assess the variation in tooth mass as a function of the interventions. The final percentage mass variation was −1.946 (±1.096) for the teeth restored with ZNO, and −1.841 (±0.918) for those restored with ZNO + GIC. Every temporary filling removal presented a negative variation in tooth mass of 0.37% for ZNO, and of 0.63% for teeth restored with ZNO + GIC, respectively.

**Conclusions:**

Successive removals of temporary restorative materials led to loss of dental structure. When comparing temporary restorations made with ZNO + GIC and ZNO, no differences in the lost mass were observed.

## 1 Introduction

Most teeth requiring endodontic treatment have already had loss of structure due to dental caries or trauma, which leads to a compromised fracture resistance. Additionally, endodontic procedures produce further structural loss, due to the removal of the pulp chamber roof and enlarging the cavity [1]. In some clinical situations, it is not possible to complete the root canal treatment and the final restoration in a single appointment; thus, temporary restorations must be employed between sessions. It is essential to ensure proper coronal sealing with temporary restorative materials to prevent marginal leakage, as it could lead to recontamination of the root canal system. The coronal sealing must be secure both between endodontic treatment sessions and between the completion of the root canal treatment and the final restoration. When selecting a temporary restorative material, clinicians must consider not only the sealing capacity but also the ease of removal of these materials between treatment sessions.

Several temporary restorative materials can be used between endodontic treatment sessions. The sealing ability of these materials presents divergent results in the literature [2, 3, 4, 5]. Therefore, clinicians may choose materials based on their personal preference, as well as based on the demands of clinical situations, such as proximal surfaces involvement in the endodontic access, aesthetic requirements, and the lifespan of the temporary restoration. The adhesion of these materials to tooth structures should be considered in the decision‐making, once they should be completely removed in between endodontic sessions and prior to the application of adhesive systems that will bond the final restoration. The proper removal of these temporary materials is difficult to achieve, especially when they adhere to dentine.

An analysis conducted on the walls of dental cavities after the removal of glass ionomer cement (GIC), used as a temporary restorative material, showed that remnants of this material were found in the samples, regardless of the removal method used. Although GIC presents good mechanical resistance to masticatory loads, excellent sealing ability, and allows the patient to maintain proper oral hygiene, its removal presents a challenge. The material's chemical adhesion to dentine and enamel, and its colour similarity to dental structures, makes it difficult to assess its complete removal, and the residual material usually left on the walls may interfere with the quality of the dentine adhesion of the final restoration [6].

Zinc oxide and calcium sulfate‐based temporary cements (ZNO) is a eugenol‐free material which sets upon contact with moisture. It is widely used by dental professionals due to its similarities with zinc oxide and eugenol cements. They present linear expansion as a result of water absorption, promoting close contact between the material and the access cavity, thus providing retention of the restoration [7]. Due to its exclusive mechanical retention, it is expected that its complete removal will be easier when compared to the removal of materials with chemical adhesion, such as GIC. The aim of this study is to evaluate the loss of tooth structure after the removal of two different temporary restorative materials between endodontic treatment sessions performed by undergraduate students.

## 2 Material and Methods

### 2.1 Preparation of Teeth and Coronal Access

Twenty human sound third molars teeth were selected from the human tooth bank of Feevale University. They were stored in distilled water prior to preparation. They were washed with running water, and calculus and organic debris adhered to the root surface were removed using Gracey curettes. Coronal endodontic access was performed using a high‐speed handpiece and a round diamond bur No. 1016 (KG Sorensen Indústria e Comércio Ltda, ES, Brazil), followed by the enlargement and smoothing of the internal walls of the pulp chamber with a high‐speed handpiece and an Endo Z bur (Endo Z No. 152 23 mm Angelus Prima Dental Ltda, PR, Brazil). The root canals were explored with K‐type files with compatible taper, accompanied by irrigation with 0.9% sodium chloride saline solution. The root canals were dried with absorbent paper points (Tanari Absorbent Paper Points, Endo Plus Comércio e Serviços Ltda, RJ, Brazil). After simulating the coronal access, teeth were stored in a desiccator at 20°C for 24 h, and their mass was then measured using a precision scale (Analytical Balance ATX 224R, Shimadzu do Brasil Ltda, SP, Brazil).

### 2.2 Temporary Restorations

After coronal endodontic access preparation and initial mass measurements, teeth were randomly divided into two groups (*n* = 10) and restored with two different temporary materials, as follows:

ZNO: teeth restored with zinc oxide and calcium sulfate‐based temporary cement (Obturador Provisório Normal, Villevie Dentalville do Brasil Ltda, SC, Brazil).

ZNO + GIC: teeth restored with an internal layer of ZNO and an external layer of GIC (Maxxion R A2, FGM Produtos Odontológicos Ltda, SC, Brazil).

All restorations were performed by a single trained operator. The floor of the pulp chamber was covered with cotton pellets, maintaining a depth of 5 mm for the insertion of temporary restorative materials. The ZNO group was restored by placing the ZNO material into the preparation using a placement spatula and covered with a cotton pellet moistened with distilled water, applying light pressure for 5 min. The ZNO + GIC group was restored with a 2 mm layer of ZNO inserted following the same protocol mentioned above, after which the cavity was dried with air jets, and the GIC was applied using a precision syringe (Aplicador de Precisão No. 2, Maquira Industria de Produtos Odontologicos SA, PR, Brazil). The GIC was covered with a layer of hydrophobic adhesive (Adper Scotchbond Multi‐Purpose, 3 M ESPE, MN, USA), which was light‐cured for 20 s (Emitter A, Schuster Comércio de Equipamentos Odontologicos Ltda, RS, Brazil).

### 2.3 Removal of Temporary Restorations and Measurement of Dental Structure Loss

One week later all temporary fillings were removed to provide a new endodontic access—the reopening. Once all material was removed, teeth were submitted to weighing protocol, and new temporary restorations were performed, as previously described. These procedures were repeated four times over successive weeks, simulating new access for follow‐up of the endodontic treatment. The reopening was performed by four predoctoral dental students coursing their final year at Feevale dental school. They received instructions detailing the materials used in the restorations. The students were provided with a high‐speed handpiece and spherical diamond burs No. 1013 and 1016 (KG Sorensen Indústria e Comércio Ltda, ES, Brazil), Endo Z bur (Endo Z No. 152 23 mm Angelus Prima Dental Ltda, PR, Brazil), clinical kit (clinical cotton plier, No. 5 explorer, No. 17 spoon excavator, and No. 5 mirror), endodontic suction, and syringe for irrigation with 0.9% saline solution. The procedures were performed under a reflector light, with teeth held in hand, without the use of any magnification tools. The students and professors who performed the removal of the temporary materials were unaware of the study's objective.

After the fourth reopening, two professors specialised in restorative dentistry finished the cavities, simulating the preparation prior to the final restoration. Teeth were air‐dried and stored in a moisture‐free environment at 20°C for 24 h, and then their mass was measured again.

### 2.4 Data Analysis

Linear regression analysis was performed to evaluate the variation in tooth mass as a function of repeated interventions for the removal of restorative materials. Two‐way ANOVA, followed by Tukey's test, was used to compare the mass variation between the different interventions and restorative materials used. A significance level of 5% was considered for all analyses.

## 3 Results

**FIGURE 1:**
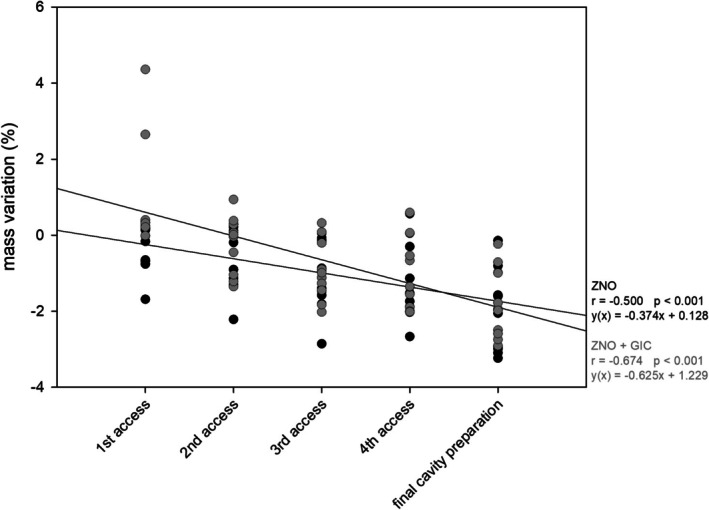
Linear regression analyses of mass variation as a function of interventions. Grey: teeth restored with ZNO + GIC; Black: teeth restored with ZNO.

Figure 1 presents the complete characterisation and distribution of mass variation values among different reopening sessions and materials, for all 10 samples evaluated in each group. After four temporary fillings' removal, the final mass variation, in percentage, was −1.946 ± 1.096 for teeth restored with ZNO and −1.841 ± 0.918 for teeth restored with ZNO + GIC. For both groups, there was a correlation between the number of reopenings and mass variation. The correlation was −0.500 and −0.674 for ZNO and ZNO + GIC, respectively. The slope was −0.374 and −0.625 for ZNO and ZNO + GIC, respectively. Therefore, with each new intervention, a negative mass variation of 0.37% is expected for teeth restored with ZNO and 0.63% for teeth restored with ZNO + GIC.

The statistical analysis showed an interaction between material and reopenings. After the cavity was finished for final restoration, there was no difference in mass variation regarding different materials. A difference was observed between groups only after the first reopening, when teeth restored with ZNO + GIC showed a positive mass variation, while those with ZNO had a negative variation. Both materials showed significant mass loss between the fourth reopening and the finishing for restoration.

## 4 Discussion

In the present study, there was a negative correlation between the number of reopenings and the loss of tooth structure, as measured by the tooth's mass variation, regardless of the temporary restorative material used. The total mass loss did not differ between groups ZNO and ZNO + GIC. There was approximately 2% of the total tooth mass after five reopenings.

The mass loss can be explained by the fact that during the removal of the restorative materials, the high‐speed diamond bur may contact the dentine walls. Inexperienced operators, such as the students who performed the coronal reopening in the present study, may have difficulty managing the high‐speed handpiece. A study evaluating students' feelings during practical training for cavity access in endodontic treatment indicated that they felt stressed during the preclinical to clinical transition, especially when they began treating real patients [8]. The training phase, accompanied by the development of manual skills and associated with anxiety, can result in preparations that are either overly invasive or insufficient to completely remove all temporary restorative material.

In the academic context, the routine of endodontic treatment usually involves multiple appointments and reopenings until the final obturation and restoration is completed. It is known that the endodontic access preparation is a moment when students experience fear and lack of confidence [9]. Often, different students participate in the treatment of a patient due to the complexity of the case, time limitations and variations in the students' technical skills. Furthermore, treatments provided by predoctoral dental students typically require more appointments than specialists [10]. These factors were the rationale background for using four alternated operators in the reopenings, as it introduced this variability into the study.

Both ZNO and ZNO + GIC are satisfactory materials/techniques for temporary restorations between endodontic treatment appointments [11]. Due to the exclusive mechanical retention of ZNO, it is plausible to expect that the ZNO group would exhibit less mass loss compared to the ZNO + GIC group, which has a chemical bond to the dental mineral structure [12], and a shade that emulates the dental tissue. Teeth from the ZNO + GIC group were restored applying the double sealing technique, using GIC as the cover restoration and the ZNO as a base material. ZNO might not have undergone complete hygroscopic expansion due to the lack of moisture after the GIC cover application. This issue might be responsible for inappropriate hardness of ZNO, making its removal easier. The use of the ‘double sealing technique’ was adopted in one of the comparison groups in this study because it is widely used in dental schools and in countries such as Saudi Arabia [11] and the United States [13]. The use of double sealing with ZNO as the base material may facilitate the removal of the temporary material, as it does not adhere to the internal walls of the preparation, making this technique suitable for use during predoctoral student training.

Unlike other studies that evaluated the presence of remnants of temporary restorative materials, or changes in the preparation walls through scanning electron microscopy [6], the present study opted for an evaluation tool based on an objective parameter—the mass loss of the tooth structure. It should be noted that the weighing method allows observing a gain in mass, which can be justified by the incomplete removal of the restorative materials. Although it was not quantified objectively, visual inspection after the successive reopenings revealed the presence of remnants of temporary restorative materials in both the ZNO and ZNO + GIC restored teeth. For teeth restored with ZNO, the greatest amount of material was observed on the internal walls of the preparation, while for teeth restored with ZNO + GIC, remnants were found on the outer edges of the cavities, which can be explained by the greater adhesion of GIC to enamel.

The results of this study, as well as the extrapolation of its findings to clinical practice, should be interpreted with caution. The conditions under which the operators removed the materials from the dental cavity did not simulate the characteristics of the oral cavity, as the teeth were manipulated freely in the operators' hands. It is reasonable to assume that in clinical practice, treated teeth present more irregular surrounding walls than those in the present study, both due to the greater difficulty of teeth's visualisation in the oral cavity and the fact that teeth may have structural losses (i.e., carious cavities) not present in the sound teeth used in this study. Furthermore, one can highlight the difficulty many patients have in opening their mouth, which limits both field of vision and access for instruments, making it more challenging to remove temporary restorative materials, resulting in greater loss of dental structure.

It should be noted that this study did not include a control group with experienced operators performing the temporary restoration removals. It is reasonable to assume that experienced practitioners, particularly those using magnification, would identify the restorative materials, glass ionomer cement and zinc oxide and calcium sulfate‐based temporary cement, more easily than students, potentially resulting in less tooth structure loss. Furthermore, in many clinical scenarios, the definitive post‐endodontic restoration involves cuspal coverage to protect the remaining tooth structure [14]. To accommodate this, the most occlusal portions of the endodontic access walls are invariably prepared during the restorative phase; consequently, any tooth structure loss in this specific region during reopening procedures is negligible. Nevertheless, measuring hard tissue loss in procedures performed by undergraduate students can serve as a valid proxy for assessing their technical skill and proficiency.

## 5 Conclusion

Successive removal of temporary restorative materials leads to the loss of tooth structure. No differences in the lost mass were observed comparing restorations made with glass ionomer cement and with zinc oxide and calcium sulfate‐based temporary cement.

## Funding

The authors have nothing to report.

## Conflicts of Interest

The authors declare no conflicts of interest.

## Data Availability

The data that support the findings of this study are available from the corresponding author upon reasonable request.
